# Antimicrobials administration time in patients with suspected sepsis: faster is better for severe patients

**DOI:** 10.1186/s40560-020-00471-2

**Published:** 2020-07-22

**Authors:** Romain Jouffroy, Benoît Vivien

**Affiliations:** SAMU de Paris, Service d’Anesthésie Réanimation, Hôpital Universitaire Necker - Enfants Malades, Assistance Publique - Hôpitaux de Paris, AP-HP.Centre and Université de Paris, Paris, France

**Keywords:** Antibiotic therapy, Sepsis, Early, Mortality, Confounder, Methodological issue

## Abstract

In a recent report, Ascuntar et al. describes the impossibility to demonstrate a significant association between early antibiotic therapy administration and mortality in sepsis patients. Nevertheless, we believe that some methodological issues deserve their conclusions. First, the objective of the authors of an early antibiotic therapy may be ambitious considering practical daily emergency department limitation. Second, most of patients included in the study appear to suffer from sepsis and not from septic shock, which limits the impact of an early and aggressive management. At last, more than a single intervention such as antibiotic therapy, sepsis treatment is now considered as based on a “bundle of care.”

To the Editor:

We read with great interest the recent paper published in the Journal by Ascuntar et al [1]. reporting the impossibility to demonstrate a significant association between early antibiotic therapy administration and mortality in sepsis patients. Undoubtedly, the authors must be complimented for this interesting study in the controversial field of early antibiotic therapy administration in case of suspected or confirmed sepsis. Nevertheless, we believe that some methodological issues deserve their conclusions.

Firstly, the database used by the authors for this analysis comes from a previous prospective study [2], in which they had concluded that an early antibiotic therapy use, i.e. within 3 h after emergency department (ED) admission, had the greatest impact on survival on septic shock patients. However, this recommendation may be ambitious considering practical daily ED limitations: on the one hand, since the 2016 SEPSIS-3 international consensus, septic shock definition requires lactatemia assessment and/or norepinephrine administration after adequate fluid expansion, which is time-consuming and frequently requires more than 3 h; furthermore, in situation of ED overcrowding, following these guidelines is much more difficult, and initial severity assessment of sepsis patients could only be based on clinical symptoms.

Secondly, in the study of Ascuntar et al., the overall hospitality mortality rate was low (11.5%), suggesting that most of patients suffered from sepsis and not from septic shock, for which mortality rate usually reaches 40 to 50% [3]. Although this point was stressed by the authors in their article [1], we think that this is the major limitation of this work, precluding any application of the results to septic shock patients.

Thirdly, considering either the 1-h or the 3-h threshold, the patients who presented with a lower systolic blood pressure were those who received early antibiotics, that is clinically fully comprehensible. Conversely, we could hypothesize that a late antibiotic administration in the less severe patients could have worsen their prognosis to the level of the most severe ones but who received an early treatment, this finally being an explanation for the absence of difference in mortality between groups.

Last but not least, more than a single intervention, sepsis treatment is based on a bundle of care, as mentioned by Ascuntar et al. in the background of their article [1]. Among many clinical and paraclinical elements, hemodynamic optimization is one of the two most important [2, 4]. Nevertheless, the authors did not use a logistic regression model to take into account the blood pressure reached, which is a major confounder [1]. Indeed, for the antibiotics to reach infected tissues, the restoration of a sufficient tissue perfusion pressure is a fundamental prerequisite [5].

Finally, we fully agree with Ascuntar et al. that early identification of sepsis, especially those at risk of unfavorable evolution, is difficult and a daily challenge. Nevertheless, we believe that while assessment of sepsis severity is a crucial step prior to treatment instauration in order to reduce sepsis mortality rate, on the opposite, indiscriminate and inappropriate use of antibiotics may be a major factor for antimicrobial resistance.

## Data Availability

Not applicable

## Abbreviations

ED: Emergency department
